# The appearance, active components, antioxidant activities, and their markers in fibrous roots of *Bletilla striata*

**DOI:** 10.1371/journal.pone.0313318

**Published:** 2024-11-11

**Authors:** Xiaoxing Li, Xinrui Sun, Ying Yang, Shangshang Yang, Jiachun Zhang, Chao Sun

**Affiliations:** 1 Guizhou University of Traditional Chinese Medicine, Guiyang, Guizhou, China; 2 Guizhou Botanical Garden, Guiyang, Guizhou, China; 3 Guiyang Public Health Treatment Centre, Guiyang, Guizhou, China; 4 Zunyi Plantation Development Service Centre, Zunyi, Guizhou, China; 5 Key Laboratory for Biodiversity Conservation in Karst Mountain Area of Southwestern China of the National Forestry and Grass land Administration, Guiyang, China; 6 Guizhou Natural Products Research Centre, Guiyang, Guizhou, China; ICAR-Directorate of Mushroom Research, INDIA

## Abstract

This study explores the potential medicinal properties of *Bletilla striata* fibrous roots by comparing their active ingredient contents and antioxidant activity with those in tubers. The study further examines the effects of growth age and origin on the distribution characteristics of medicinal properties in fibrous roots and identifies biomarkers that distinguish these characteristics. The Δ E value of fibrous roots significantly increases with age and is significantly influenced by origin; both the age and origin also significantly affect militarine content and antioxidant activity; a positive correlation exists between appearance quality, active ingredients, and antioxidant activity. The total phenolic content and antioxidant activity of fibrous roots significantly exceed those of tubers. The militarine content in both 1-year-old and 2-year-old fibrous roots complies with the 2020 edition of the Chinese Pharmacopoeia, suggesting that the fibrous roots possess medicinal potential. Additionally, this study demonstrates that the OPLS-DA model can differentiate 1-year-old and non-1-year-old fibrous roots of *Bletilla striata* and distinguish between their origin.

## 1.Introduction

In recent years, a global embrace of a “back to nature” concept and the role of Chinese medicine in addressing the COVID-19 pandemic have presented the Chinese medicine industry with a strategic opportunity. However, extreme weather has affected both the production [1, 2] and quality [3] of Chinese herbal medicines, leading to market shortages. The scarcity of herbal resources is partly because traditional practices only use specific medicinal parts of plants; other parts are discarded at harvest. However, because these discarded parts may contain valuable medicinal components, their disposal not only represents waste, but it may degrade soils [4] and create a continuous cropping obstacle [5]. Exploring the medicinal potential of “non-essential” parts of plants could expand the medicinal utility of herbs, increase supply, and mitigate environmental degradation.

The Chinese ground orchid *Bletilla striata* (Thunberg) Reichb.f. occurs mainly in China, Korea, Myanmar, and Japan [6]. For over 1500 years, this species has been extensively used in traditional Chinese medicine [7]. The Divine Husbandman’s Classic of the Materia Medica describes its taste as being bitter, sweet, astringent, and slightly cold, and attributes its properties to the lung, liver, and stomach meridians. It is known for its hemostatic and astringent qualities and its ability to alleviate swelling and promote muscle regeneration [8]. Traditional applications include treating hemoptysis, hematemesis, traumatic bleeding, sores, swelling, and chapped skin [9].

Not only has widespread, destructive excavation of wild *B*. *striata* occurred, but seeds of this species are small and challenging to germinate in natural conditions. This has led to declines in the natural resources of this species. Therefore, to meet increased market demand, cultivation is essential and presently represents the primary source of medicinal plants. The tuber of *B*. *striata* is the traditional part of the plant used for medicinal purposes; the fibrous root and above-ground parts of the plant have conventionally been considered non-medicinal and have been discarded in the soil during harvest. However, the root of *B*. *striata* contains chemical components similar to those in the tubers, with some compounds even occurring at significantly higher concentrations [10]. Components in these roots possess antimicrobial [10], antiulcer [11], anticarcinogenic [12, 13], and antioxidant [14] properties. In addition, mean ratios of root weight (g) to tuber weight (g) in Yunyan District (0.396), Bozhou District (0.191), and Shibing County (0.583) (unpublished data) suggest that 1 t of tubers could yield 191 kg of roots, which represents both significant waste and environmental pollution.

To date, research on the roots of *B*. *striata* has focused on chemical composition and antioxidant activity. As a perennial Chinese medicinal herb, the quality of *B*. *striata* tubers is influenced by their age [15, 16] and geographic origin(hereinafter “origin”) [17, 18]. However, the impact of neither age nor origin on the quality and antioxidant activity of roots has been reported. Because the appearance of Chinese herbal medicines can indicate their quality, we explore: 1) relationships between the appearance of *B*. *striata* roots and their active ingredients and antioxidant activity; 2) differences in active ingredient content and antioxidant activity based on age and origin; and 3) markers associated with variations in age and origin.

## 2. Materials and methods

### 2.1. Materials

No human participants, specimens or tissue samples, or vertebrate animals, embryos, or tissues were involved in this study.

Reagents used in this research and their sources include: 1,1-diphenyl-2-trinitrophenylhydrazine (DPPH), 2,2’-diazobis (3-ethylbenzothiazoline-6-sulfonic acid) (ABTS), and 2-phenyl-4,4,5,5-tetramethylimidazoline-3-oxo-1-oxide (PTIO), supplied by Nanjing Jiancheng Bioengineering Institute, with purities of ≥ 98%. HPLC-grade acetonitrile and analytical-grade sulfuric acid, anthrone, anhydrous sodium carbonate, phenol, and ethanol were provided by Chongqing Platinum Strontium Titanium Technology Co., Ltd. Gallic acid, vitamin C reference standards, glucose reference standards, etc., were sourced from Guizhou Dida Technology Co., Ltd., with purities of ≥ 98%. The militarine reference substance was sourced from Chongqing Platinum Strontium Titanium Technology Co., Ltd., with a purity of ≥ 98%.

### 2.2. Plant materials

In 2023, *B*. *striata* tubers were harvested from Yunyan District (YY) of Guiyang City (106°42′E, 36°24′N), Bozhou District (BZ) of Zunyi City (106°33′E, 27°38′N), and Shibing County (SB) of Qiandongnan Prefecture (108°12′E, 26°58′N). Tubers had been planted for three years. Species identification was confirmed by Wang Yong, an associate researcher at the Guizhou Botanical Garden. Tubers were categorized into three grades (1–3) based on the number of years they had been in the soil. Grade 1 tubers represented new growth from the current year that had been in the soil for one year. Tubers of different growth ages were interconnected by structures known as “bridge rods.” Fibrous roots were excised from tubers of each growth age and processed and ground to produce root powder samples. These samples were bagged, sealed, and stored in a dry place for future use.

### 2.3. Assessing root visual quality

An NH300 portable spectrophotometer (San’enshi Technology Co., Ltd.) was used to inspect fibrous root appearance. The whiteboard, included in instrument accessories, was used for calibration and adjustment [19]. Each sample was subjected to three replicate measurements, for which color values (ΔL, Δa, Δb, and ΔE) were recorded. ΔL represents the difference between the sample and correction value, indicating the relative brightness (dark or light) of a sample compared with the whiteboard; a value of “0” indicates a complete sample match, whereas positive and negative values indicate a sample is lighter or darker than the whiteboard, respectively. Δa and Δb similarly represent the differences between a sample and a correction value. Δa indicates the color of a sample (red or green); a value of “0” indicates a neutral color, whereas positive and negative values indicate redness or greenness, respectively. Δb indicates the color of the tested sample (yellow or blue); a value of “0” indicates a neutral color, whereas positive and negative values indicate yellowness or blueness, respectively. E represents the total color difference, and ΔE is calculated as follows: ΔE = [(ΔL)^2^+(Δa)^2^+(Δb)^2^]1/2.

### 2.4.Determining polysaccharide contents

A 100.0 mg sample of root powder [20] was transferred to a round-bottomed flask, and 75.0 mL of distilled water was added; the flask and contents were weighed, then placed on an electric heating mantle and gently simmered for 2 h. After cooling, the solution was topped up with distilled water to its original weight, mixed well, and filtered. Using a pipette, 10.0 mL of the filtrate was collected, to which 40 mL of anhydrous ethanol was added, mixed well, and the solution left to stand overnight. The sample was then centrifuged at 8000 rpm for 10 min, the supernatant decanted, and the precipitate dissolved in a suitable volume of hot distilled water. After cooling, the solution was transferred to a 50-mL volumetric flask, diluted to the mark with distilled water, and mixed thoroughly. A 2 mL sample of standard and test solutions was pipetted into a 10.0 mL EP tube, to which 5.0 mL of a 0.2% sulfuric acid-anthrone solution [21] was added and mixed well. The solution was heated in a boiling water bath for 10 min, mixed well, then quickly immersed in an ice water bath for 10 min. Absorbance was measured at 625 nm by UV spectroscopy. Linear regression was performed using glucose mass concentration (mg/mL) as the abscissa and absorbance value as the ordinate to calculate sample polysaccharide contents.

### 2.5.Militarine content determination

A 1 g sample of root powder was subjected to ultrasonic extraction with 100 mL of 50% ethanol [16]. Following filtration, the resultant extract was analyzed via high-performance liquid chromatography using an ACE Excel 5 C18-AR (5 μm, 4.6 mm × 250 mm) chromatographic column, with the mobile phase consisting of an acetonitrile-0.1% phosphoric acid solution [16] (22:78) for isocratic elution; other parameters included: flow rate 1.0 mL/min, detection wavelength 223 nm, injection volume 10 μL, and column temperature at 25°C.

### 2.6. Measuring total phenolic content

Total phenols were extracted as per the method in Section 2.5. A 1.0 mL sample was pipetted from a series of standards and samples into a 10 mL EP tube, to which 2.5 mL of phenol solution was added and mixed well, followed by 2.5 mL of sodium carbonate solution (15%), and then 4.0 mL of ultrapure water and mixed well. The solution was incubated in a 40°C water bath for 60 min, then allowed to cool for 20 min before measuring absorbance at 770 nm [22]. Total phenol concentrations for samples were based on the standard curve.

### 2.7. Measuring vitamin C equivalent antioxidant capacity

Following the method of Xinyi et al. [23], the vitamin C equivalent antioxidant capacity (VCEAC) of roots was measured. After dispensing 50 μL of Vc control solutions of varying concentrations into a 96-well plate, 200 μL of DPPH solution was added, mixed well, and incubated in the dark at room temperature for 20 min. The absorbance of each well was measured at 517 nm to construct a standard curve relating DPPH absorbance to Vc concentration:

$$Y519nm=0.008X+0.0068,\;R^{2}=0.9997$$

The VCEAC value of the 50% ethanol extract (mg Vc/g sample)was calculated as follows:

VCEAC(mg/g)=(Y−0.068)/0.008×V/M

where Y is the absorbance of the DPPH solution, V is the volume of the extraction solvent in mL, and M is the mass of the extract (g).

A 20 μL of Vc reference solution of varying concentrations was pipetted into a 96-well plate, followed by the sequential addition of 200 μL of ABTS·working solution. The plate was well agitated and incubated in the dark at room temperature for 6 min. The absorbance of each well was assayed at 734 nm to create a standard curve relating ABTS-solution absorbance to Vc concentration:

$$Y734\;nm=0.0086X-0.044,\;R^{2}=0.999$$

To determine the VCEAC value (mg Vc/g sample) of the 50% ethanol extract, the following formula was used:

VCEAC(mg/g)=(Y+0.0444)/0.0086×V/M

where Y represents the absorbance of the ABTS·solution, V denotes the volume of the extraction solvent used (mL), and M signifies the mass of the extract (g).

A 100 μL sample of Vc reference working solution of varying concentrations was pipetted into a 96-well plate, followed by the sequential addition of 100 μL of PTIO·working solution. Samples were mixed well and incubated in darkness for 2 h. Absorbance of each well was measured at 557 nm to construct a standard curve relating PTIO-solution absorbance to Vc concentration:

$$Y557\;nm=0.008X+0.0608,\;R^{2}=0.999$$

The VCEAC value of the 50% ethanol extract (mg Vc/g sample) was calculated using the following formula:

VCEAC(mg/g)=(Y−0.0608)/0.008×V/M

where Y denotes the absorbance of the PTIO·solution, V represents the volume of extraction solvent added (mL), and M signifies the mass of the extract (g).

### 2.8. Measuring free radical scavenging abilities

#### 2.8.1. DPPH radical scavenging ability

Based on Xinyi et al. [23], we used an adapted method to determine DPPH radical scavenging ability. Specifically, the blank group used a mixture of 50% ethanol (500 μL) and DPPH working solution (2.0 mL). The sample group used antioxidant activity sample solutions (each 500 μL) and DPPH working solution (2.0 mL) from various growth ages and origins. For the control group, antioxidant activity sample solutions (each 500 μL) and anhydrous ethanol (2.0 mL) from different growth ages and origins were used. All mixtures were thoroughly shaken in a stoppered test tube and incubated at room temperature in the dark for 20 min. After incubation, absorbance was measured at 517 nm using the blank solvent as a reference; the following absorbance values were recorded: A0 (blank group), A1 (sample group), and A2 (control group).

#### 2.8.2. ABTS radical scavenging ability

Following the methodology of Xinyi et al. [23], we implemented suitable modifications. The blank group comprised 200 μL of 50% ethanol and 2.0 mL of ABTS working solution; the sample group comprised 200 μL of antioxidant activity sample solution (from various ages and origins) and 2.0 mL of ABTS working solution; and the control group comprised 200 μL of antioxidant activity sample solution (from different origins) and 2.0 mL of 80% ethanol. After mixing all solutions in stoppered test tubes, they were incubated at room temperature in darkness for 6 min. After the reaction, absorbance was measured at 734 nm, with values recorded as A0 (blank group), A1 (sample group), and A2 (control group).

#### 2.8.3. PTIO radical scavenging ability

PTIO radical scavenging ability was determined following a slightly modified method of Renqiang et al. [24]. The three sample groups were the blank (1.0 mL phosphate buffer solution + 1.0 mL PTIO solution), the sample (1.0 mL of test solution + 1.0 mL of PTIO solution), and the control (1.0 mL of test solution + 1.0 mL of phosphate buffer solution). After thorough mixing, the reactions were incubated in darkness for 2 h. Absorbance of each group was measured at 557 nm and recorded as A0 (blank group), A1 (sample group), and A2 (control group). Using Vc as a standard, a standard curve was constructed relating concentration to scavenging efficiency to calculate the Vc-equivalent concentration for each group in accordance with the following equation:

Scavengingefficiency(%)=A0(A1−A2)/A0×100%

### 2.9. Statistical analysis

Statistical analyses were performed based on three replicated experiments. One-way analysis of variance (ANOVA) was performed using GraphPad Prism 9, with the least significant difference calculated at a 95% confidence level. Pearson’s correlation analysis was performed using SPSS 27.0.1 eigenvalues. OPLS-DA analysis was performed using SIMCA 14.1, and images were processed using Adobe Illustrator 2023.

## 3. Results and discussion

### 3.1. Appearance characteristics of roots

#### 3.1.1. The effect of age on root color

With increased root age ΔL, Δa, and Δb decrease, and ΔE increases (Table 1). Significant differences in ΔE values occur among ages. Because a link exists between herb color and quality [25, 26], variations in ΔE among ages may indicate differences in root quality.

**Table 1:** Color values of *Bletilla striata* roots aged 1–3 years.

Color value	Age 1 year	Age 2 year	Age 3 year
ΔL	−50.08	−56.98	−60.70
Δa	10.32	10.00	9.68
Δb	21.22	19.28	17.63
ΔE	55.41^c^	61.04^b^	64.01^a^

a, b, and c indicate significant differences in ΔE between ages (p < 0.05).

#### 3.1.2. Effect of origin on root color

The order of ΔL values for roots changes with age: 1 y, SB > BZ > YY; 2 y, YY > SB > BZ; and 3 y, BZ > YY (Table 2). Additionally, Δa and Δb values for 1- and 2-y-old roots are ranked YY > SB > BZ. For ΔE, rankings of values for 1-y-old roots were YY > BZ > SB, with values for roots from YY being significantly higher than those from SB; for 2-y-old roots, this rank was BZ > YY >SB. Because there were few 3-y-old roots from SB, color values for this age could not be measured. The Δa, Δb, and Δ E values of 3-y-old fibrous roots all show that YY>BZ.

**Table 2:** Color values of *Bletilla striata* roots from different regions by age.

Geography	Color value	1 year	2 year	3 year
YY	ΔL	−50.66	−55.71	−62.20
	Δa	11.51	10.79	11.32
	Δb	22.57	20.69	17.90
	ΔE	56.69^ab^	60.42^a^	65.78^a^
BZ	ΔL	−51.40	−58.39	−59.45
	Δa	9.57	9.38	8.32
	Δb	20.20	18.29	17.40
	ΔE	56.08^bc^	61.97^a^	62.55^a^
SB	ΔL	−46.46	−56.30	
	Δa	9.84	9.92	
	Δb	21.01	18.88	
	ΔE	51.95^c^	60.20^a^	

Superscript letters (a, b, c) indicate significant differences in ΔE values for the same age (*p*< 0.05) between regions.

### 3.2. Active ingredients in roots

#### 3.2.1. The effect of age on active ingredients in roots

**Fig 1:**
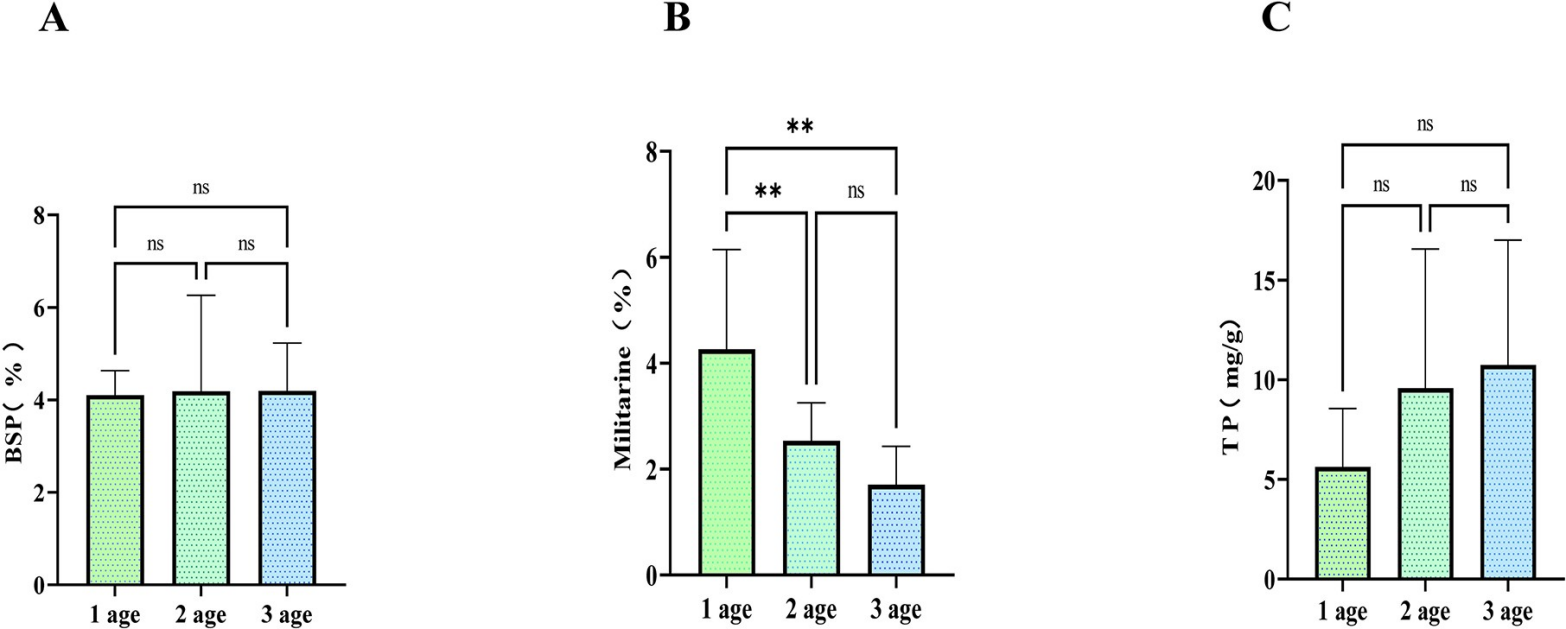
Effect of age on active ingredient contents in *Bletilla striata* roots: A) polysaccharide (BSP) content; B) militarine content; and C total phenolic (TP) content. ns,*p*> 0.05; * *p*<0.05;** *p*< 0.01.

The polysaccharide content in roots increased with age, yet the disparity among different ages was not significant (Fig 1A). This could be attributed to the lower levels of polysaccharide synthesis metabolites in fibrous roots [27], resulting in slower annual accumulation. Conversely, the synthesis and accumulation of polysaccharide anabolites was most pronounced in tubers, with significant differences across ages. A progressive decline in polysaccharide content in tubers occurred from ages 1–3 y, with a highly significant disparity among age groups (S1A Fig). This may be because with increased age, nutrients are transferred from older to younger tubers through “bridging rods” [28]. Polysaccharides, considered primary metabolites [29], may also gradually convert to secondary metabolites after 2 y of planting to support physiological requirements for growth, development, and defense. This process likely promotes the synthesis and accumulation of polysaccharides in 1-y-old tubers.

The militarine content in roots gradually decreased with age (Fig 1B), with contents in 1-y-old roots significantly higher than in 2- and 3-y-old roots. However, the militarine content in 2-y-old roots was slightly higher than that of 3-y-old roots. The militarine contents in 1-y-old roots were 1.88× higher than those in 1-year-old tubers. Conversely, the militarine content in 2- and 3-y-old roots was 0.566× and 0.367× lower than that in 2 and 3-y-old tubers, respectively (S1B Fig). This inverse trend suggests a potential transfer of militarine from roots to tubers. In addition, according to the 2020 edition of the Chinese Pharmacopoeia [9], the militarine content should be no less than 2.0% in *B*. *striata* medicinal herbs or 1.5% in *B*. *striata* decoction pieces. Militarin contents in 1- and 2-y-old *B*. *striata* roots exceeded 2.0%, and although that in 3-y-old roots was slightly below 2.0% it was higher than 1.5%, indicating that *B*. *striata* roots have medicinal potential.

The total phenol content of roots increased with age (Fig 1C), but differences were not significant; this indicates that growth can promote the accumulation of total phenols in fibrous roots. Numerous studies have demonstrated the total phenol content of *B*. *striata* roots to exceed that of tubers; we report total phenol contents of 1-y-old roots to be highly significantly different from total phenol contents of 1-y-old tubers, and for total phenol contents of 2- and 3-y-old roots to be significantly higher than 2- and 3-y-old tubers. While total phenols in tubers are considered to be of medicinal value, roots have significantly higher total phenol contents and therefore also represent a medicinal resource (S1C Fig).

#### 3.2.2. The effect of origin on effective constituents of roots

**Fig 2:**
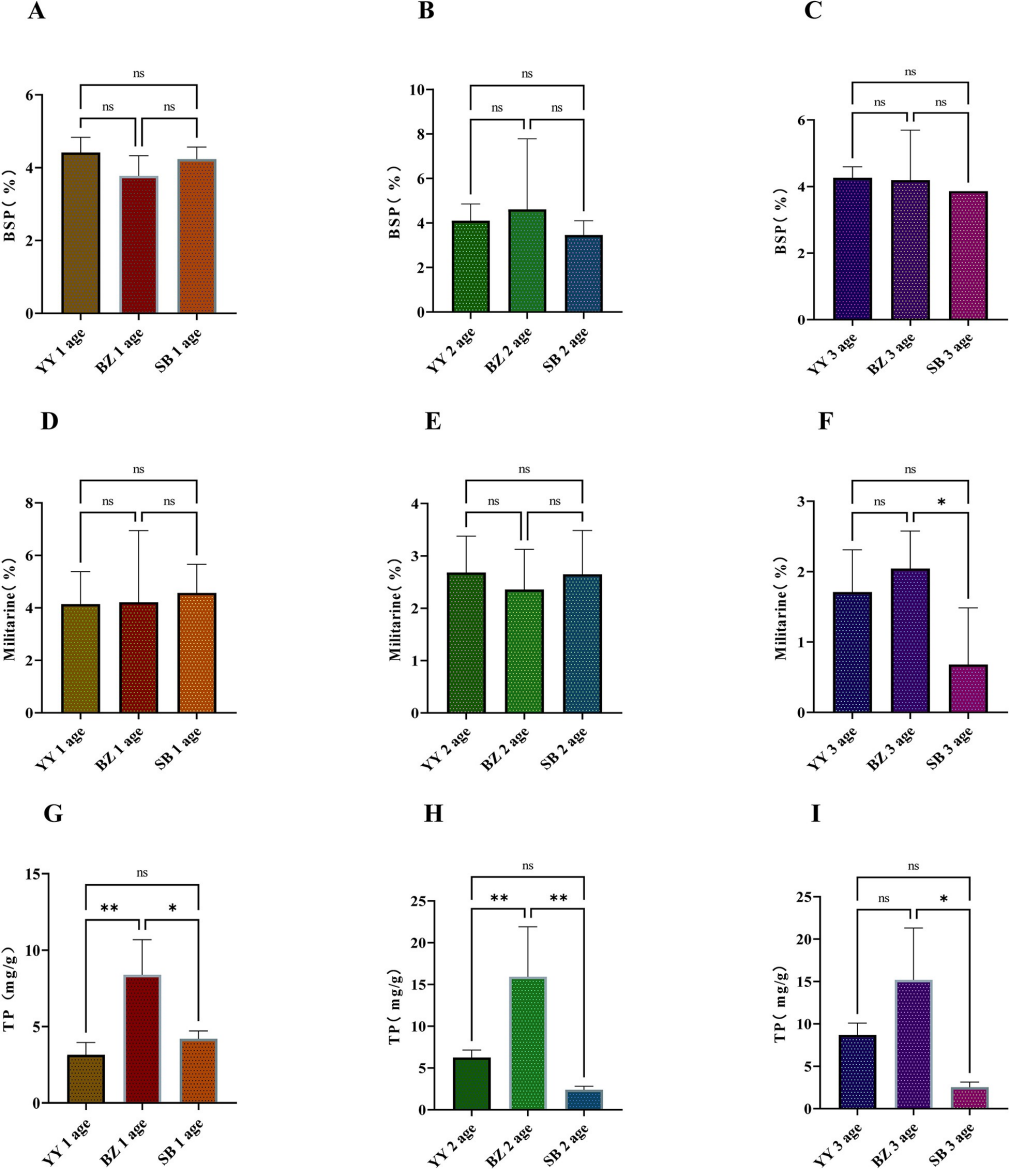
Effect of origin on active ingredient contents in *Bletilla striata* roots: A–C polysaccharide content; D–F militarine content; and G–I total phenolic content. YY, Yunyan District; BZ, Bozhou District; SB, Shibing County. ns,*p*> 0.05, * *p*< 0.05, ** *p*< 0.01.

Differences in polysaccharide content occurred in roots of different ages from the three locations at which they were collected. The content of polysaccharides in 1-y-old roots was highest at YY and lowest at BZ (Fig 2A); for 2-y-old roots, this order was BZ > YY > SB (Fig 2B), and for 3-y-old roots, YY > BZ > SB (Fig 2C). Militarine content in roots of different ages varied in YY, BZ, and SB, with contents in 1-y-old roots highest at SB and lowest at YY (Fig 2D); for 2-y-old roots, this rank was YY > SB > BZ (Fig 2E), and for 3-y-old roots, BZ > YY > SB. The militarine content of 3-y-old roots from BZ was significantly higher than from SB (Fig 2F). Root total phenol varied with age in YY, BZ, and SB, and was highest at BZ; total phenol in 1-y-old roots was significantly higher at BZ than at SB; contents from BZ were extremely significantly higher than total phenol from YY, and contents from SB were slightly higher than those from YY (Fig 2G). Significantly more total phenol occurred in 2-y-old roots from BZ than from SB and YY; total phenol contents in roots from YY were only marginally higher than those from SB (Fig 2H). For 3-y-old roots, total phenol contents were significantly higher from BZ than SB, following the order BZ > YY > SB (Fig 2I). Origin affected neither polysaccharide nor militarine contents of 1- and 2-y-old roots, but differences were significant for 3-y-old roots.

Accumulation of secondary metabolites is influenced by environmental factors such as light, temperature, soil moisture, fertility, and salinity [30]. Changes in these factors can alter secondary metabolite contents in plants, even when other factors remain constant. A positive correlation exists between polysaccharides and the abundance of fungal genera *Cystofilobasidium* and *Thielavia*, and Zn^2+^, Al^3+^, and Ca^2+^, and a negative correlation exists between the abundance of fungal genera *Phoma*, *Penicillium*, *Fusarium*, and Na+ [31]. Moisture also significantly affects polysaccharide accumulation in *B*. *striata* [32]. The microbiome in sandy clay soils enhances militarine accumulation in *B*. *striata* tubers, while that of sandy loam soils inhibits its accumulation [33]. Additionally, a significant negative correlation occurs between total phenol of *B*. *striata* and soil polyphenol oxidase, phosphatase, and pH, and a significant positive correlation exists with organic matter [34]. Accordingly, soil fertility affects the active ingredient contents of *B*. *striata*, and variations in soil fertility caused by geographic differences [35] may explain differences in effective components.

### 3.3. Antioxidant activity of roots

#### 3.3.1 The effect of age on root antioxidant activity

**Fig 3:**
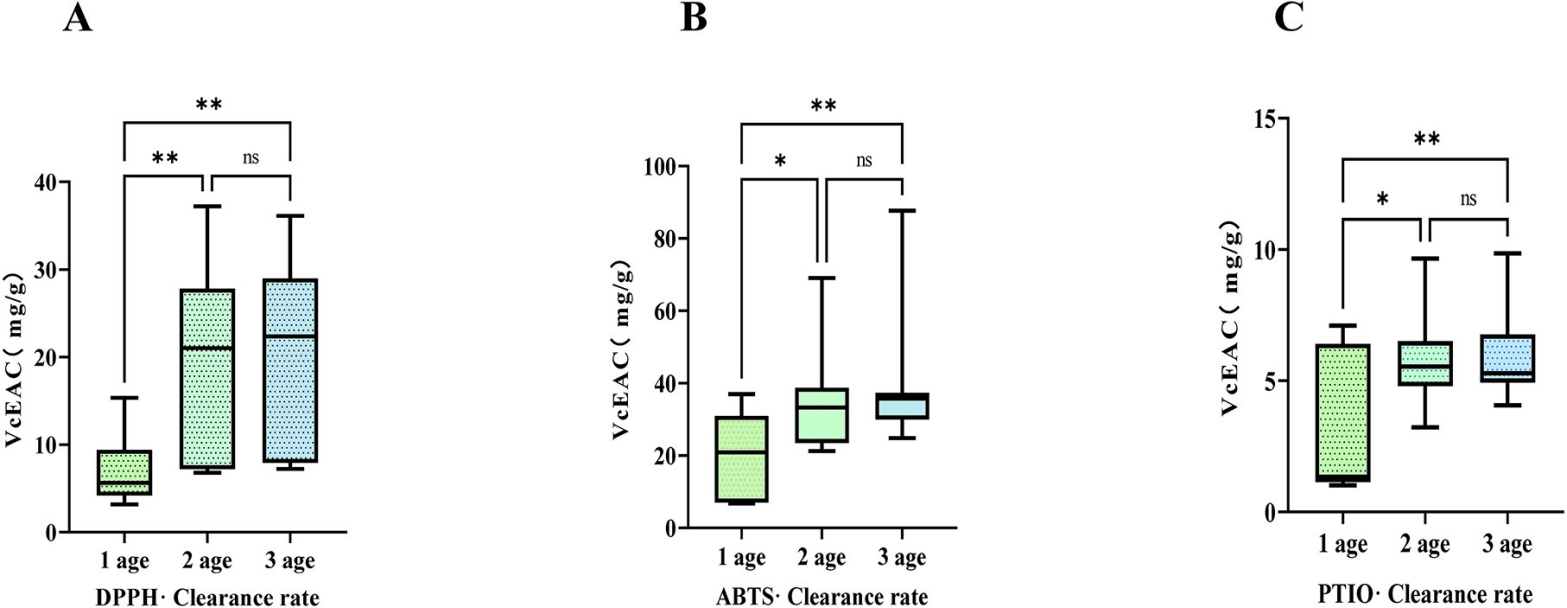
Effect of age on vitamin C equivalent antioxidant capacity (VcEAC) of *Bletilla striata* roots on: A)1,1-diphenyl-2-trinitrophenylhydrazine(DPPH) scavenging ability; B)3-ethylbenzothiazoline-6-sulfonic acid(ABTS)·scavenging ability; and C)2-phenyl-4,4,5,5-tetramethylimidazoline-3-oxo-1-oxide (PTIO)·scavenging ability. ns, *p*> 0.05, * *p*< 0.05, ** *p*< 0.01.

The ability of roots to scavenge free radicals increased with age (Fig 3). The trend in Vc-equivalent values for DPPH free radical scavenging increased with age. Notably, the Vc-equivalent value for DPPH free radical scavenging by roots at age 1 y was significantly lower than that 2- and 3-y age. Additionally, the Vc-equivalent value for DPPH free radicals scavenging by roots at 2 y was lower than that for 3 y age (Fig 3A). The Vc-equivalent values of ABTS free radical removal by roots increased with age. The Vc-equivalent values of ABTS free radical removal by 1-y-old roots were significantly lower than those of 2-y-old roots and even more significantly lower than those of 3-y-old roots (Fig 3B). The Vc-equivalent values of PTIO free radical removal by 1-y-old roots were significantly lower than those of 2-y-olds and highly significantly lower than those of 3-y-olds. In contrast, the Vc-equivalent values of PTIO free radical removal by 3-y-old roots were slightly higher than those of 2-y-old roots (Fig 3C). The impact of age on antioxidant activity was most obvious between 1 and > 1-y-old roots, with no significant difference in antioxidant activity apparent between roots of 2- and 3-y age.

The Vc equivalent value of DPPH free radical removal by roots exceeded that of tubers. Specifically, at the age of 1 y, the Vc equivalent value of DPPH free radical removal by roots exceeded that of tubers. Moreover, at ages 2 and 3 y, the Vc equivalent values of DPPH free radical removal by roots were significantly higher than those of tubers (S2A Fig). Additionally, the Vc-equivalent values of ABTS free radical removal by roots were significantly higher than those of tubers (S2B Fig). Vc equivalent values for PTIO free radical scavenging by roots significantly exceeded those of tubers (S2C Fig). Polysaccharides and total phenol were the main components responsible for antioxidant activity, and antioxidant activity correlated positively with polysaccharide and total phenol contents. Despite the higher polysaccharide content in tubers, their antioxidant capacity was lower than that of roots, mainly because the in vitro antioxidant assay used a total phenol extract, extracted with 50% ethanol. Polysaccharides of *B*. *striata* are more polar and less soluble in 50% ethanol, resulting in higher antioxidant activity in roots compared with tubers. The antioxidant activity of roots was stronger than that of tubers, indicating their strong antioxidant properties. Therefore, *B*. *striata* roots represent an unexploited, valuable medicinal, and potentially commercial resource.

#### 3.3.2. Effects of origin on root antioxidant activity

**Fig 4:**
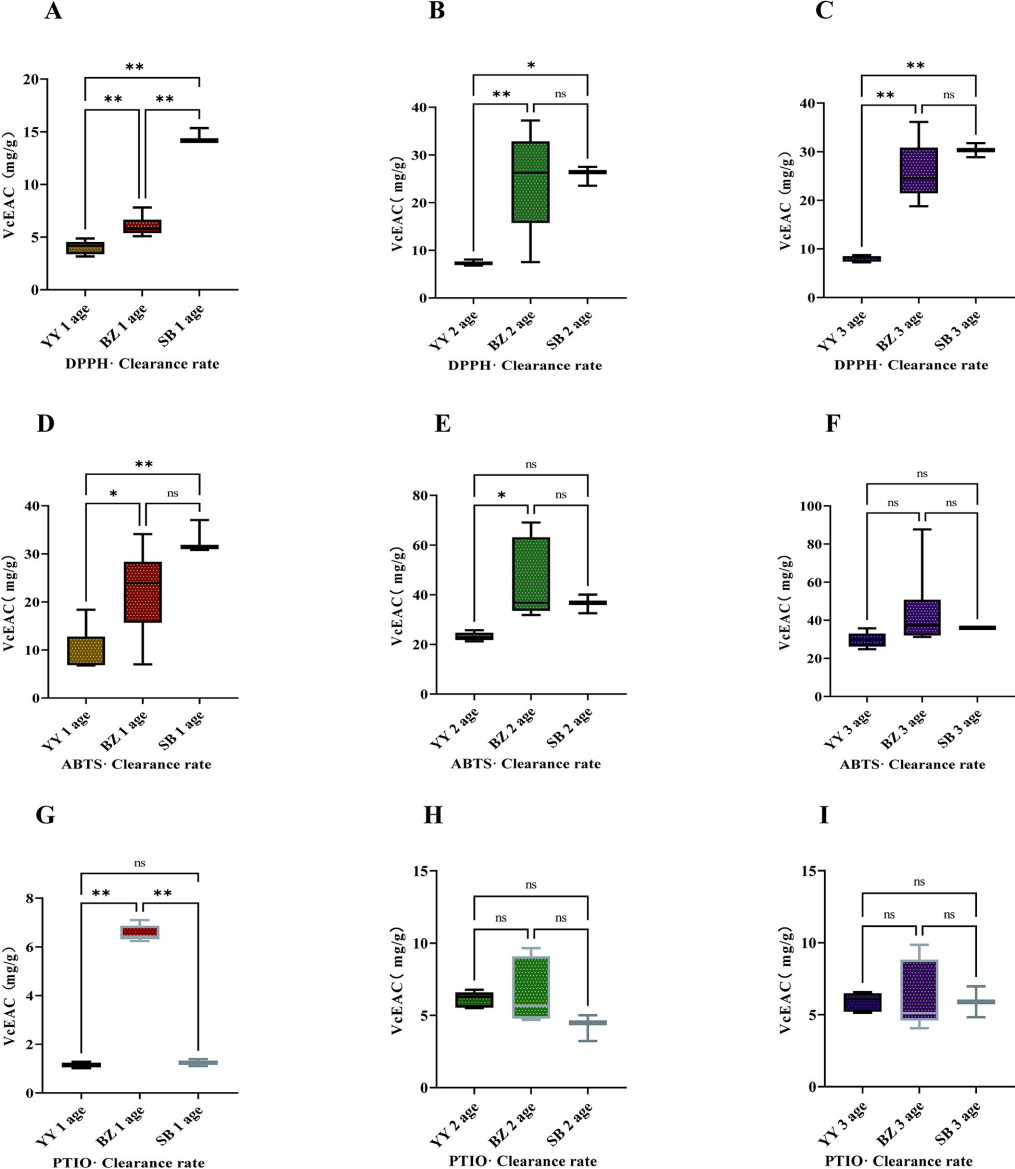
Effect of origin on antioxidant activity of *Bletilla striata* roots on: A–C, 1,1-diphenyl-2-trinitrophenylhydrazine (DPPH)·scavenging activity; D–F, 3-ethylbenzothiazoline-6-sulfonic acid (ABTS)·scavenging activity; and G–I,2-phenyl-4,4,5,5-tetramethylimidazoline-3-oxo-1-oxide (PTIO) scavenging activity. ns, *p*> 0.05; * *p*< 0.05; ** *p*< 0.01.

The Vc-equivalent values of DPPH free radical removal by roots varied across ages for YY, BZ, and SB; the highest Vc-equivalent values occurred in SB. At 1-y age, Vc-equivalent values of DPPH free radical removal by roots were highest in SB and lowest in YY, and there were significant differences in the Vc equivalent values among locations (Fig 4A). The Vc-equivalent values of DPPH free radical removal by 2-y-old roots were significantly lower in YY compared with BZ and SB, with SB showing higher values than BZ (Fig 4B). Similarly, Vc-equivalent values of DPPH free radical removal by 3-y roots were significantly lower in YY than in BZ and SB, with SB again having higher values than BZ (Fig 4C). Variation in ABTS free radical scavenging capacity occurred in roots of different ages in YY, BZ, and SB. The Vc-equivalent values for ABTS free radical scavenging by 1-y-old roots in YY were significantly lower than those from BZ and extremely significantly lower than SB (Fig 4D). The Vc-equivalent values for ABTS free radical removal by 2- and 3-y-old roots were highest from BZ and lowest from YY. Only Vc-equivalent values for ABTS free radical removal by 2-y-old roots from BZ were significantly higher than those from YY (Fig 4E and 4F). Differences in PTIO free radical scavenging ability occurred among roots of various ages in YY, BZ, and SB. The Vc-equivalent values of PTIO free radical scavenging by 1-y-old roots from BZ were significantly higher than those from YY and SB, while those of 1-y-old roots from SB were also higher than those from YY (Fig 4G). VC-equivalent values for PTIO free radical scavenging by 2-y-old roots were highest from BZ and lowest from SB (Fig 4H); for 3-y-old roots, values were highest from BZ and lowest from YY (Fig 4I).

### 3.4. Correlation between appearance, active ingredients, and antioxidant activity of roots

Numerous studies have demonstrated strong associations between the color of herbal medicines and their active ingredients [25, 26] and antioxidant activities [36]. Pearson’s correlation analysis results are presented in Table 3. No correlation was apparent between appearance and polysaccharide contents of roots. The correlation between ΔL and Δb values and militarine content was strong and positive, suggesting that higher ΔL and Δb values corresponded to increased militarine content. There was also a highly significant positive correlation between ΔE and total phenol content, indicating that the higher the ΔE value, the higher the total phenol content. The ΔE value of roots was significantly positively correlated with DPPH free radical clearing ability and highly significantly positively correlated with ABTS free radical and PTIO free radical clearing abilities, indicating that the higher the ΔE value, the greater the ability of roots to clear DPPH, ABTS, and PTIO free radicals. The ΔE values correlated positively with total phenol and antioxidant activity. Additionally, antioxidant activity was affected by age, so differences in ΔE between ages may be because of variation in antioxidant activity.

**Table 3:** Correlations between appearance, active ingredients, and antioxidant activity in *Bletilla striata* roots.

Norm	Polysaccharide	Militarine	Total phenol	ΔL	Δa	Δb	ΔE	DPPH	ABTS	PTIO
polysaccharide	1									
militarine	−0.069	1								
total phenol	0.521**	−0.348	1							
ΔL	−0.286	0.598**	−0.603	1						
Δa	0.029	0.135	−0.482	0.096	1					
Δb	−0.349	0.471**	−0.709	0.796**	0.502**	1				
ΔE	0.288	−0.584	0.546**	−0.989	0.036	−0.709	1			
DPPH	0.283	−0.280	0.623**	−0.446	−0.578	−0.635	0.370*	1		
ABTS	0.355*	−0.236	0.767**	−0.555	−0.520	−0.748	0.479**	0.810**	1	
PTIO	0.248	−0.333	0.647**	−0.545	−0.325	−0.558	0.500**	0.361*	0.455**	1

* *p*< 0.05

** *p*< 0.01.

### 3.5. Principal component analysis

**Fig 5:**
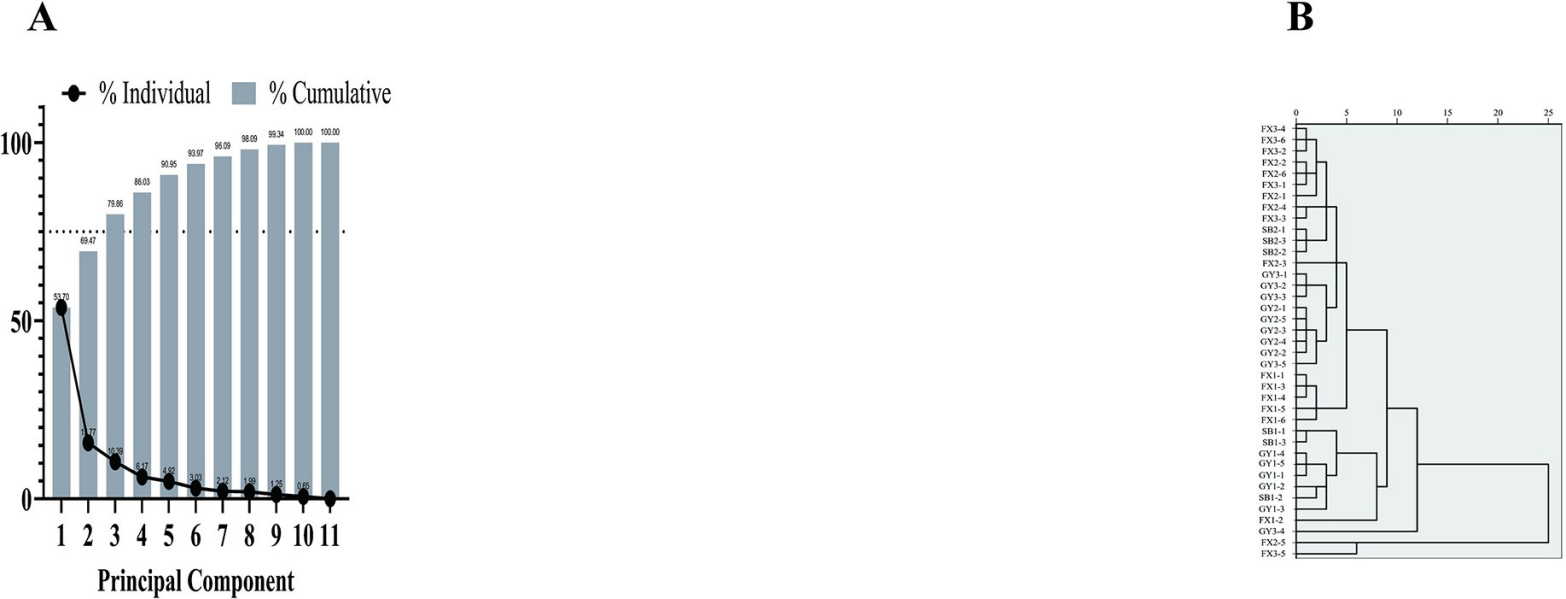
Results of principal component analysis: A) principal component fragmentation plot; B) clustering dendrogram between indicators.

The composite quality of roots of various ages and origins was calculated using GraphPad Prism 9 (Fig 5; Tables 4, 5). Eigenvalues of the first three principal components exceeded 1, with a cumulative variance contribution rate of 79.86% (Fig 5A). The eigenvalue of PC1, 5.90, explained 53.70% of the variance. Table 4 reports age, militarine, total phenols, ΔL, Δb, ΔE, and DPPH free radical, ABTS free radical, and PTIO free radical scavenging capacities to have high loadings on PC1. The eigenvalue of PC2, 1.73, explained 15.77% of the variance; Δa has a substantial loading on PC2 (Table 4). The eigenvalue of PC3, 1.14, explained 10.39% of variance; polysaccharides had a higher loading on PC3 (Table 4). The decision to replace the original 11 variables with 3 principal components (PC1–3) was based on their ability to effectively condense the information from all indicators. Composite scores of PC1, PC2, and PC3 (Table 5) revealed the top 10 scores (BZ3-5, BZ2-3, BZ3-6, BZ3-3, BZ3-4, BZ2-4, BZ3-2, BZ2-5, BZ2-6, and BZ1-5) were all located in BZ. This outcome suggests that the composite qualities of roots from BZ exceeded those from YY and SB.

**Table 4:** Principal component loading results.

Sports event	Principal component		
	PC1	PC2	PC3
growing age	0.767	−0.404	0.282
polysaccharide	0.399	0.171	−0.832
militarine	−0.592	0.444	−0.266
total phenol	0.838	0.289	−0.216
ΔL	−0.878	0.415	0.050
Δa	−0.447	−0.673	−0.469
Δb	−0.906	−0.073	−0.037
ΔE	0.818	−0.511	−0.110
DPPH	0.727	0.449	0.122
ABTS	0.808	0.408	−0.026
PTIO	0.683	0.034	−0.056

**Table 5:** Combined scores for *Bletilla striata* roots of different origin and age.

Origin	Serial number	1 y old	Ranking	2 y old	Ranking	3 y old	Ranking
YY	1	−1.537	37	−0.597	31	−0.183	20
	2	−0.845	33	−0.344	25	0.020	17
	3	−1.110	36	−0.458	28	−0.313	24
	4	−2.191	39	−0.505	29	−0.447	27
	5	−2.015	38	−0.894	34	−0.666	32
BZ	1	−0.292	23	0.074	16	0.612	11
	2	−0.991	35	0.553	13	1.096	7
	3	−0.082	18	1.489	2	1.304	4
	4	−0.137	19	1.146	6	1.302	5
	5	0.637	10	1.043	8	2.506	1
	6	−0.568	30	0.801	9	1.410	3
SB	1	−0.277	22	0.223	15		
	2	−0.408	26	0.333	14		
	3	−0.244	21	0.556	12		

To comprehensively analyze and evaluate similar component information more intuitively and reflect the differences in quality indicators between different production areas and different growth ages, raw data for the 11 quality indexes from 39 *B*. *striata* roots were standardized. Systematic clustering using the squared Euclidean distance as a metric and intergroup connection as a clustering method (Fig 5B) revealed two groups (BZ2-5 and BZ3-5, and the remaining 37 samples) at a squared Euclidean distance > 15. However, these clusters did not effectively differentiate age or origin. Therefore, we opted for orthogonal partial least squares discriminant analysis (OPLS-DA) to assess the classification model for root age and origin.

### 3.6. Classification of the roots of *Bletilla striata*

**Fig 6:**
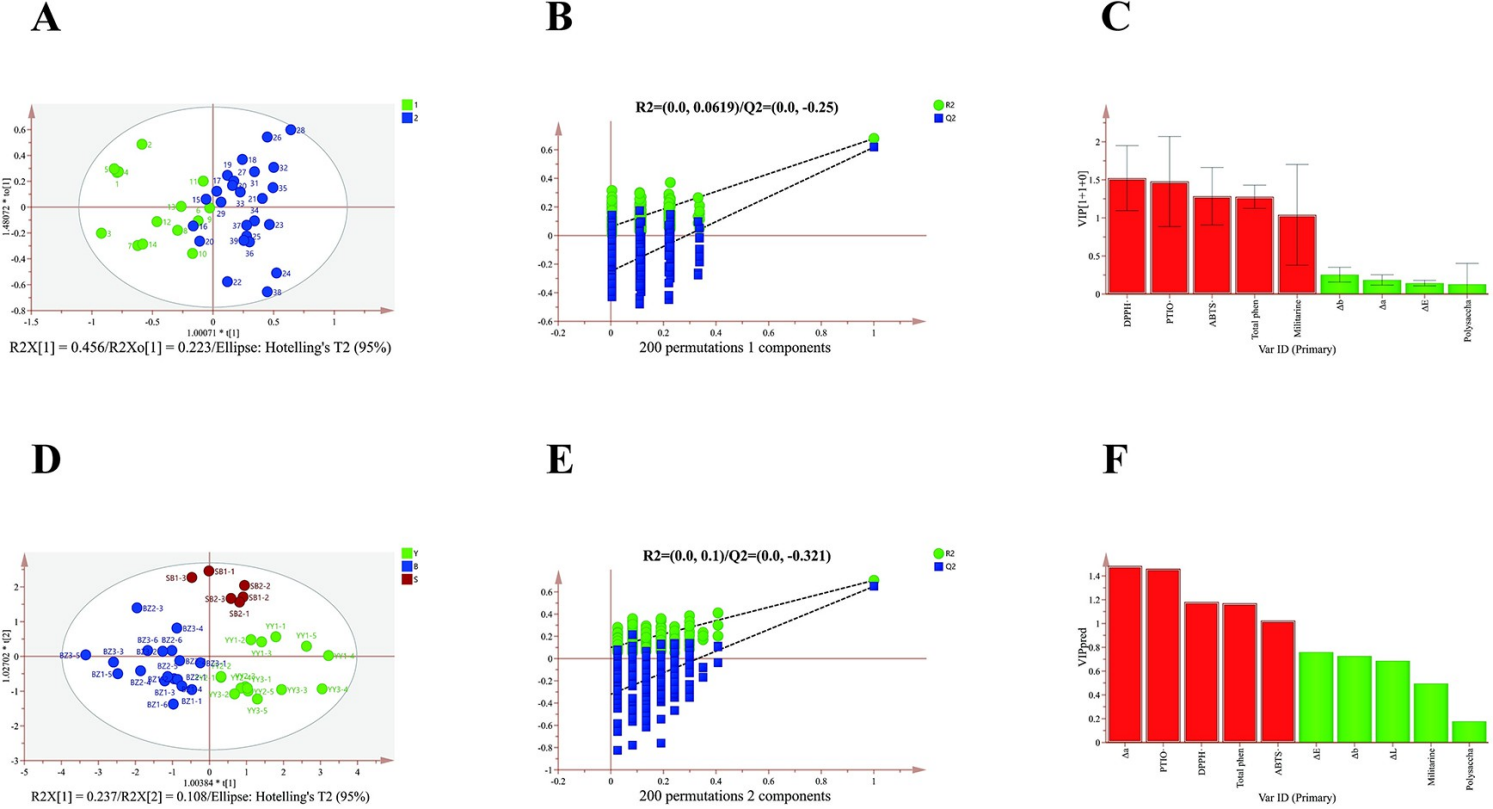
Results of OPLS-DA analysis on *Bletilla striata* roots. A) OPLS-DA scores, B) 1-component 200 permutation test, and C) VIP value plots for 1-y-old(1) and > 1-y-old (2) models; and D) OPLS-DA scores, E) 2-component 200 permutation test, and F) VIP value plots for the model of roots from different origins.

The influence of age on active ingredients and antioxidant activity was most apparent between 1-y-old and > 1-y-old roots. Consequently, we adopt age as the dependent variable and consider root appearance, active ingredients, and antioxidant activity to be independent variables. Using SIMCA 14.1 software, an OPLS-DA discriminant model was constructed for root age (Fig 6A and 6C) and origin (Fig 6D and 6F). Accumulated explanatory power parameters measured by the OPLS-DA model for the growth age of roots were 0.679 for R2X and 0.678 for R2Y, and 0.758 for R2X and 0.702 for R2Y for their origin. Both models demonstrate stable and reliable predictive power parameters, with Q2 values of 0.618. The distinction between age 1 and > 1-y-old roots is evident (Fig 6A). The scatter plot of OPLS-DA scores reveals the locations where roots were collected to occur across three regions, with significant differences between them where complete separation was achieved (Fig 6D).

The OPLS-DA models for age and origin were tested with 200 permutations. The intersection of the Q2 regression line with the vertical axis for both models was below 0, indicating the reliability and absence of over fitting in constructed models (Fig 6B and 6E).The Variable Projection Importance (VIP) value serves as a crucial index for screening differential compounds, with a higher VIP indicating a greater impact on group differences. Using a VIP value >1 as the threshold, results are depicted in Fig 6C. From the model of different root ages, five characteristic difference indicators were derived: DPPH free radical scavenging capacity (1.522) > PTIO free radical scavenging capacity (1.478) > ABTS free radical scavenging capacity (1.284) > total phenol (1.277) > militarine (1.040). These markers exert varying degrees of influence on the classification of 1-y and > 1-y-old roots. The model analyzing root origin identified five indicators of characteristic differences: Δa (1.484) > PTIO free radical scavenging capacity (1.460) > DPPH free radical scavenging capacity (1.182) > total phenol (1.170) > ABTS free radical scavenging capacity (1.024) (Fig 6F). These indicators significantly impact the origin classification of roots.

## 4. Conclusion

*Bletilla striata* root appearance is influenced by age and origin and correlates positively with militarine, total phenol, and antioxidant activity. This indicates that root appearance can be used to indicate medicinal quality. The polysaccharide content of roots is unaffected by age and origin, but total phenol is influenced by origin. While root total phenol content is not affected by age, it does increase with age, with the highest contents found in 3-y-old roots. We report higher total phenol contents in roots than tubers, with a militarine content meeting standards outlined in the 2020 edition of the Chinese Pharmacopoeia. Additionally, root antioxidant activity exceeds that of tubers, suggesting that roots represent an unexploited medicinal resource.

While the highest quality roots were collected in BZ, the two clusters produced by systematic cluster analysis did not differentiate samples based on age or origin. An OPLS-DA discriminant model used to analyze disparities between root age and origin identified five indices (DPPH free radical, PTIO free radical, and ABTS free radical scavenging capacities, and total phenol and militarine) to be cumulative markers that could differentiate 1-y-old from > 1-y-old roots, and five indicators (DPPH free radical, PTIO free radical, and ABTS free radical scavenging capacities, and Δa and total phenol) that could differentiate their origin. These results improve our understanding of the potential medicinal value of *B*. *striata* roots, at what age and where the highest quality roots may be found, and how their medicinal quality may be visually assessed.

## Supporting information

S1 Fig The impact of different parts on active ingredients. T represents tuber; FR represents fibrous root; A represents the polysaccharide content in tuber; B represents the militarine content in different regions; and C represents the total phenolic content in different regions.
(TIF)

S2 Fig The influence of different parts on antioxidant capacity. T represents tuber; FR represents fibrous root; A represents the variation in the capacity of different parts to scavenge DPPH free radicals; B represents the variation in the capacity of different parts to scavenge ABTS free radicals; and C represents the variation in the capacity of different parts to scavenge PTIO free radicals.
(TIF)

S1 Dataset (XLSX)
