# Supplementary figures and images for: The appearance, active components, antioxidant activities, and their markers in fibrous roots of Bletilla striata
Source: PLoS One. 2024 Nov 11;19(11):e0313318. doi: 10.1371/journal.pone.0313318 (PMC11554209; doi:10.1371/journal.pone.0313318)

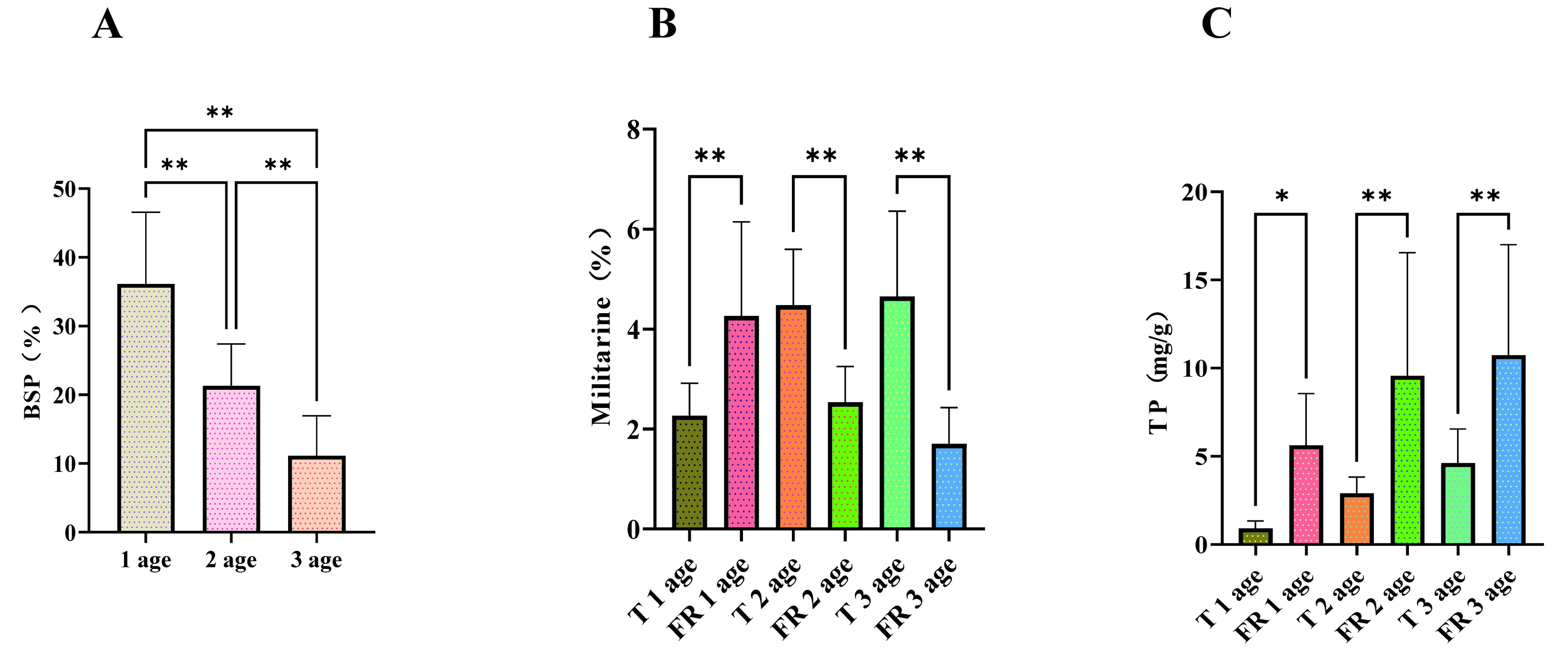

Supplement: S1 Fig — T represents tuber; FR represents fibrous root; A represents the polysaccharide content in tuber; B represents the militarine content in different regions; and C represents the total phenolic content in different regions. (TIF) [file pone.0313318.s001.tif]

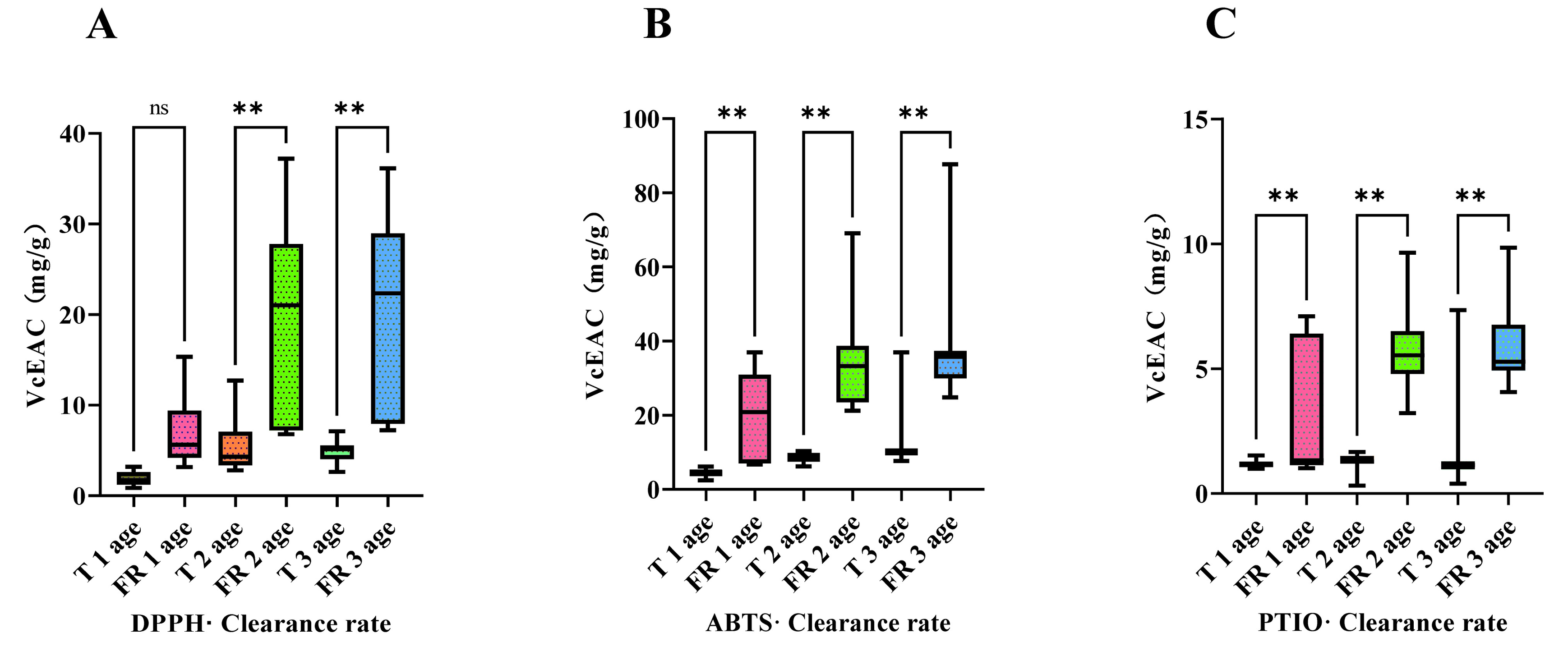

Supplement: S2 Fig — T represents tuber; FR represents fibrous root; A represents the variation in the capacity of different parts to scavenge DPPH free radicals; B represents the variation in the capacity of different parts to scavenge ABTS free radicals; and C represents the variation in the capacity of different parts to scavenge PTIO free radicals. (TIF) [file pone.0313318.s002.tif]
